# ApoD Mediates Binding of HDL to LDL and to Growing T24 Carcinoma

**DOI:** 10.1371/journal.pone.0115180

**Published:** 2014-12-16

**Authors:** Sten Braesch-Andersen, Lena Beckman, Staffan Paulie, Makiko Kumagai-Braesch

**Affiliations:** 1 Mabtech, Nacka Strand, Sweden; 2 Metabolism Unit, Department of Endocrinology, Metabolism and Diabetes, Karolinska Institute at Karolinska University Hospital Huddinge, Stockholm, Sweden; 3 CLINTEC, Division of Transplantation Surgery, Karolinska Institutet, Stockholm, Sweden; Heart Research Institute, Australia

## Abstract

Apolipoprotein (Apo) D is an important protein produced in many parts of the body. It is necessary for the development and repair of the brain and protection from oxidative stress. The purpose of this study was to investigate the extent to which apoD interacts with lipoproteins in human plasma. By using detergent-free ELISA, we show that immobilized monoclonal antibodies against apoD very efficiently bind to low density lipoprotein (LDL) from plasma; this binding is as equally efficient as binding to an anti-apoB monoclonal antibody. Adding detergent to the plasma inhibited the binding, suggesting that the binding is dependent on the presence of intact lipoprotein particles. Reversing the system by using immobilized anti-apoB revealed that the affinity of apoD for LDL is rather low, suggesting that multiple bindings are needed for a durable connection. Biosensor experiments using purified lipoproteins also showed that purified apoD and high density lipoprotein 3 (HDL3), a lipoprotein fraction rich in apoD, were both able to bind LDL very efficiently, indicating that the HDL3-LDL interaction may be a physiological consequence of the affinity of apoD for LDL. Furthermore, we found that apoD increases the binding of HDL to actively growing T24 bladder carcinoma cells but not to quiescent, contact-inhibited, confluent T24 cells. This result is especially intriguing given that the T24 supernatant only contained detectable levels of apoD after growth inhibition, raising the possibility that alternating the expression of apoD and a putative apoD-receptor could give direction to the flow of lipids. In the current paper, we conclude that apoD mediates binding of HDL to LDL and to growing T24 carcinomas, thereby highlighting the importance of apoD in lipid metabolism.

## Introduction

ApoD is a lipocalin that mainly associates with HDL3 (small dense High Density Lipoprotein) [1] in human plasma and to a lesser degree with LDL (low density lipoprotein) and VLDL (very low density lipoprotein). The predicted molecular weight of apoD is 20 kD, but, due to glycosylation, the protein migrates as a 28–30 kD band in SDS-PAGE [2], [3]. As a lipocalin, apoD has an eight-stranded antiparallel beta-sheet barrel-like structure forming a hydrophobic pocket [4], which has been shown to bind to a variety of hydrophobic ligands such as progesterone, pregnenolone and arachidonic acid [2]. ApoD is ubiquitously expressed throughout the body, notably by glial cells in the brain and by fibroblasts [2]. Apart from in the brain, high concentrations of apoD can be found in the spleen, testes and mammary cysts [5].

The importance of apoD was revealed when it was shown that Drosophila flies, transfected with human apoD, displayed significantly increased life expectancy [6]. This increased life expectancy was suggested to be due to reduced lipid peroxide accumulation [6]. Consistent with this hypothesis, apoD has been shown to confer resistance to oxidative stress [7]. ApoD has also been shown to be important in several pathophysiological situations, and it can mediate nerve repair both in the brain and in the periphery [8], [9]. During repair of peripheral nerves, the local concentration of apoD can increase up to 500 times . The concentration of apoD also increases in the brain, and to some extent in plasma, as a result of ageing and of brain disorders such as Alzheimer's disease, stroke or brain infections [2], [11], [12]. Using a mouse model of lethal atherosclerotic coronary heart disease, Tsukumoto et al. [13] showed that the protective effect of apoD extends to the heart. The over-expression of ApoD also suppresses inflammation induced by Corona virus-induced encephalitis [14].

Apo D, in addition to being a potential marker of brain disorders, appears to act in the brain to protect from oxidative stress and inflammation and to distribute lipids [15]. ApoD has also been shown to bind amyloid-β-plaques and may be instrumental, along with apoE and apoJ, in removing amyloid-β from the brain [15]. As with apoE, certain alleles of apoD have been linked to an increased risk of developing Alzheimer's disease [16], [17], [18]. There are no known cases of total apoD deficiency in humans, but apoD knockout mice display learning disabilities and impaired locomotion [7].

Even though apoD is associated with lipoprotein particles, its role in lipid metabolism has not been clearly established. However, apoD has been implicated in triglyceride metabolism [19], and mice lacking apoD have increased triglyceride levels in postprandial plasma samples [20].

ApoD also acts as a transporter of lipid hormones [2]. There is evidence that apoD is involved in LCAT regulation [21], [22], and LCAT, apoA1 and apoD have been suggested to form a functional entity with a stoichiometry of 1∶1∶2 [23]. Furthermore, Bhatia et al. [24] showed that apoD display antioxidant properties and was able to reduce lipid hydroperoxides to inert hydroxides. Unlike several other apolipoproteins, apoD is expressed by a variety of cells and is upregulated upon growth arrest (quiescence) in cell lines [25], [26], [27], [28], [29]. Addition of purified apoD to vascular smooth muscle cells inhibits PDGF-BB-induced proliferation [29] and induces apoptosis in colorectal carcinoma cells [30].

An important function of HDL particles is to deliver lipids to LDL and VLDL particles, and it is reasonable to assume that this interaction is facilitated/regulated in some way. Miller et al. [31] showed that HDL and LDL mutually inhibited each other when binding to fibroblasts. Their results indicate either that LDL and HDL compete for the same receptors on the fibroblasts or that the interaction between HDL and LDL in solution interfered with the binding to the fibroblasts.

In the present study we have investigated the potential role of apoD in the interaction between lipoproteins and between lipoproteins and cells. Our results show that apoD may provide an important link in the interaction between HDL and LDL particles and between HDL particles and cells, thus, apoD may serve as an important regulator of lipid trafficking.

## Materials and Methods

### 2.1 Antibodies, lipoproteins and cell lines

Monoclonal antibodies (mAb) to apoD were generated by immunizing mice with human recombinant apoD. Antibody specificity was confirmed by immunoprecipitation followed by mass spectrometry, and three mAbs (D544, D201 and D263) were selected and used in the study. Other mAbs used were the LDL20 and LDL17 mAbs against human apoB, the E981 mAB against apoE, the H219 and H464 mAbs against apoH, the J29 and J84 mAbs against apoJ and two control mAbs, the 7-B6-1 mAb against human IFN-γ and the IL4-I mAb against IL-4 (all from Mabtech, Nacka Strand, Sweden). A positive control antibody against human thioredoxin reductase (TrxR1), which was used for immunostaining the T24 cells, was a kind gift from Anders Rosén, University of Linköping.

Recombinant apoD was obtained from BioVendor (Modrice, Czech Republic). To increase the solubility, this recombinant apoD was modified at the following positions: Trp99-His, Cys116-Ser, Ile118-Ser. Other modifications include Leu23-Pro, Pro133-Val and Asn134-Ala. For more information, see the BioVendor homepage. Purified ApoA1, LDL, VLDL, HDL, HDL2 and HDL3 were obtained from the Academy Bio-Medical Company Inc. (Houston, TX, USA). Native apoD was purified from plasma by affinity chromatography. In short, 6.5 ml EDTA/aprotinin plasma was centrifuged first at 300 g for 10 minutes to remove cells, followed by centrifugation at 14000 g for 3 minutes to remove platelets and particles. Irrelevant mouse antibodies were added to the plasma to a final concentration of 90 µg/ml in order to block HAMA-activity (human anti-mouse antibodies). The plasma was then diluted 4x with PBS Tween20 for a final concentration of 0.025% Tween20 and passed at a flow rate of 0.2 ml/min over a 5 ml affinity-column coupled with the anti-apoD mAb D544. The column was washed with PBS, and apoD was eluted with 0.7% HAC and immediately flash-frozen (S1 Figure).

The bladder carcinoma cell line T24 (ATCC, Manassas, USA) was cultured in either DMEM or RPMI 1640 medium (Gibco, BRL, Life Technology Ltd. Paisley, Scotland) with 10% FBS (HyClone, Thermo Scientific).

### 2.2 Blood samples

Finger blood was obtained from consenting healthy volunteers (approved by Regionala Etikprövningsnämnden Stockholm, 2006/227-31/1) by piercing the top of the finger with a lancet and recovering 20 µl blood with an adjustable pipette and a sterile tip. The blood was immediately transferred to Eppendorf tubes containing 480 µl dilution buffer (PBS, 0.5 mM EDTA, 0.2% BSA and 20 µg/ml irrelevant mouse IgG), and cells were removed by spinning for 3 minutes at 800 g. Finally, the supernatant was transferred to a new tube and centrifuged at 14000 g for 3 minutes to remove platelets. For the purpose of this publication, we arbitrarily calculated the volumes of the finger blood plasma based on the observation that the finger blood samples were approximately half blood cells and half plasma. Except when stated differently in the figure legends the finger blood plasma came from the same donor, although repetitions of the finger blood experiments were partly conducted using different donors.

### 2.3 Immunoassays

ELISA assays to measure apoD, apoA1 and apoB (Mabtech) were performed according to the manufacturer's instructions. Dual-specific ELISA, capable of detecting direct or indirect interactions, was performed using antibodies with different target specificities for capture and detection. To not disturb the integrity of the complexes, the incubation and washing buffers did not contain detergent. In short, the coating antibodies were diluted to 2 µg/ml in PBS with 0.02% azide, except for mAb LDL20, which was used at 1 µg/ml, and 100 µl per well were added to 96-well ELISA plates (Costar, Corning, NY, USA). After a two hour incubation at room temperature, the plates were blocked with 0.1% BSA in PBS azide (incubation buffer) for a further two hours at room temperature or in the refrigerator overnight. The plates were then washed in an automatic ELISA washer (ELx50, BioTek Instruments, Winooski, VT, USA), using PBS as the wash buffer. Purified apolipoproteins or finger blood plasma was diluted in the incubation buffer, and 100 µl/well were added to the ELISA plates for a one hour incubation. After washing with PBS, the biotinylated antibodies were diluted in the incubation buffer to 1 µg/ml, except for LDL20-biotin, which was used at 0.5 µg/ml, and antibodies were added to the plates. Following a one hour incubation, plates were again washed, Streptavidin-ALP (Mabtech), diluted in the incubation buffer, was added, and the plates were incubated for 1 hour. After further washing, the plates were developed by adding the substrate pNPP (para-Nitrophenylphosphate, S0942, Sigma-Aldrich, St. Louis, MO, USA) in a 30 mM, pH 9.0, Tris-HCl buffer supplemented with 2 mM MgCl^2^. The readings were performed at 405 nm in a Thermo max microplate reader (Molecular Devices, Ca. USA).

### 2.4 ApoD-mediated cell binding of HDL

T24 bladder carcinoma cells were seeded onto 48-well plates (Costar) at varying concentrations to generate both confluent and non-confluent cell layers. To obtain approximately 50% confluent cultures, cells were seeded 24 hours before the experiment, whereas confluent cells were seeded 5 days before to obtain achieve full contact (confluency) and a cobblestone-like pattern. The cells were washed three times with serum-free RPMI buffered with 20 mM HEPES. Next, 200 µl of 1 µg/ml HDL were added to each well, with or without 100 ng/ml of apoD or apoA1, in an incubation buffer consisting of RPMI with HEPES and 0.1% BSA. After a 1 hour incubation at room temperature, the cells were washed three times with 500 µl of RPMI and 200 µl of PBS with 0.1% BSA and 0.05% Tween20 was added. After 10 minutes, the supernatants were transferred to a low-binding 96-well V-shaped polypropylene plate (no. 651201, Greiner Bio one, Germany) and centrifuged at 1100 g for 5 minutes to remove non-solubilized material. Finally, 100 µl from each well were collected, and the concentration of ApoA1 was determined by ELISA.

### 2.5 Biosensor analysis

The binding between apoD and lipoprotein particles was assessed using a Blitz biosensor (ForteBio, Pall Life Science, Menlo Park, CA, USA). This biosensor measures mass by how it changes optical layer thickness and thus the interference patterns of reflected light. For more details, see the Pall Life Science homepage. In brief, biotinylated mAb D544 (anti-apoD) and control mAbs E981 (anti-apoE) and 7-B6-1 (anti-IFNγ isotype control for D544) were added to Streptavidin-coated biosensor surfaces, followed by rinsing and addition of purified apoD and/or lipoprotein particles. PBS with 0.1% BSA and 0.02% sodium azide was used as a buffer, and both the rinsing and loading steps were performed using a volume of 250 µl. Antibodies were loaded at a concentration of 5 µg/ml, apoD at 2 µg/ml and HDL2, HDL3 and LDL at 100 µg/ml. The loading time was 3 minutes for antibodies and 5 min for lipoproteins, and the rinsing time was 30 seconds.

### 2.6 ApoD production by T24 carcinoma cells

T24 cells were grown in DMEM supplemented with 10% FBS in 25 cm^2^ cell culture flasks (Costar). Supernatants from the confluent cultures, corresponding to approximately 2.5 million cells per flask, and non-confluent cultures, corresponding to 1–1.5 million per flask (70–80% confluent), were collected 24 hours after changing the media. For each condition, the supernatants from three flasks were probed four times and tested for apoD content via apoD ELISAs (Mabtech), with a detection limit of approximately 0.1 ng/ml. The same cells were also harvested and used for flow cytometry (see below).

### 2.7 Flow cytometry

For intracellular staining, T24 cells were removed from 25 cm^2^ tissue culture flasks by washing once with 5 ml PBS containing 1 mM EDTA, followed by a 10 minute incubation at 37°C with 3 ml of the same buffer. Detached cells were transferred to a 15 ml centrifuge tube and resuspended with a pipette to obtain a single cell suspension. The cells were washed once in PBS and, after sedimentation at 250 g for 10 minutes, the cell pellet was re-suspended in 100 µl PBS and 3 ml of a freshly prepared buffer of PBS with 0.5% TritonX100 and 2% paraformaldehyde. After 10 minutes fixation at room temperature, the cells were resuspended by adding 9 ml PBS and then pelleted again by centrifugation at 1000 g for 5 minutes. The supernatant was removed, and the remaining reactive groups were inactivated by addition of 1% glycine in PBS, azide, and 0.1% BSA. After another 20 minutes, the cells were pelleted again at 1000 g for 5 minutes and then re-suspended in 1 ml of PBS, azide, 0.1% BSA. Cell staining was performed in V-shaped 96-well plates (type 651201, Greiner Bio One, Frickenhausen, Germany) using 250K cells/well, and primary antibodies were added to a final concentration of 2 µg/ml. After a 30 minute incubation at room temperature, the cells were washed twice with PBS before adding a secondary FITC-conjugated rabbit F(ab)2 anti-mouse IgG antibody (F0313, Dako, Glostrup, Denmark) diluted 1/100 in PBS, azide, 0.1% BSA. After incubation for 30 min, the cells were again washed, followed by analysis in in a Guava EasyCyte flow cytometer (Millipore, Billerica, MA, USA).

### 2.8 Immunocytochemical staining

Cells were seeded onto glass microscope slides placed in petri dishes to maintain humidity. After incubation for 2 days (non-confluent) or 9 days (confluent) in a 37°C incubator, the cells were washed in PBS and fixed for 20 min in PBS containing 2.7% paraformaldehyde and 0.3% TritonX100. The cells were then washed with PBS and blocked with PBS containing 0.1% BSA and 1% glycine for 30 min, followed by washing and incubation with primary antibodies at room temperature. In addition to the anti-apoD mAb D544, a negative isotype-control mAb (IL4-1) and a positive control mAb (TrxR1) were used (all at 5 µg/ml). After 1 h, the cells were washed and incubated with a secondary rabbit anti-mouse-IgG FITC-conjugated F(ab)2 (DAKO) antibody (1∶100) for another hour. After washing, cell nuclei were stained for 5 min with DAPI (1 µg/ml) (Pierce) and, after a final wash, were covered with a cover glass. Cells were analyzed and photographed using a Leica DMRB fluorescent microscope equipped with a Nikon Coolpix 4500 camera (shutter speed: FITC, 2 sec; DAPI, 1/30 sec).

### 2.9 Statistical analysis

Results are presented as means with standard deviation. Statistical analysis was performed with the GraphPad Prism 6 program using the Mann-Whitney-U-test. Differences were considered statistically significant if p-values were less than 0.05.

## Results

### 3.1 Detergent-free dual-specific ELISA reveals lipoprotein interactions

**Figure 1:**
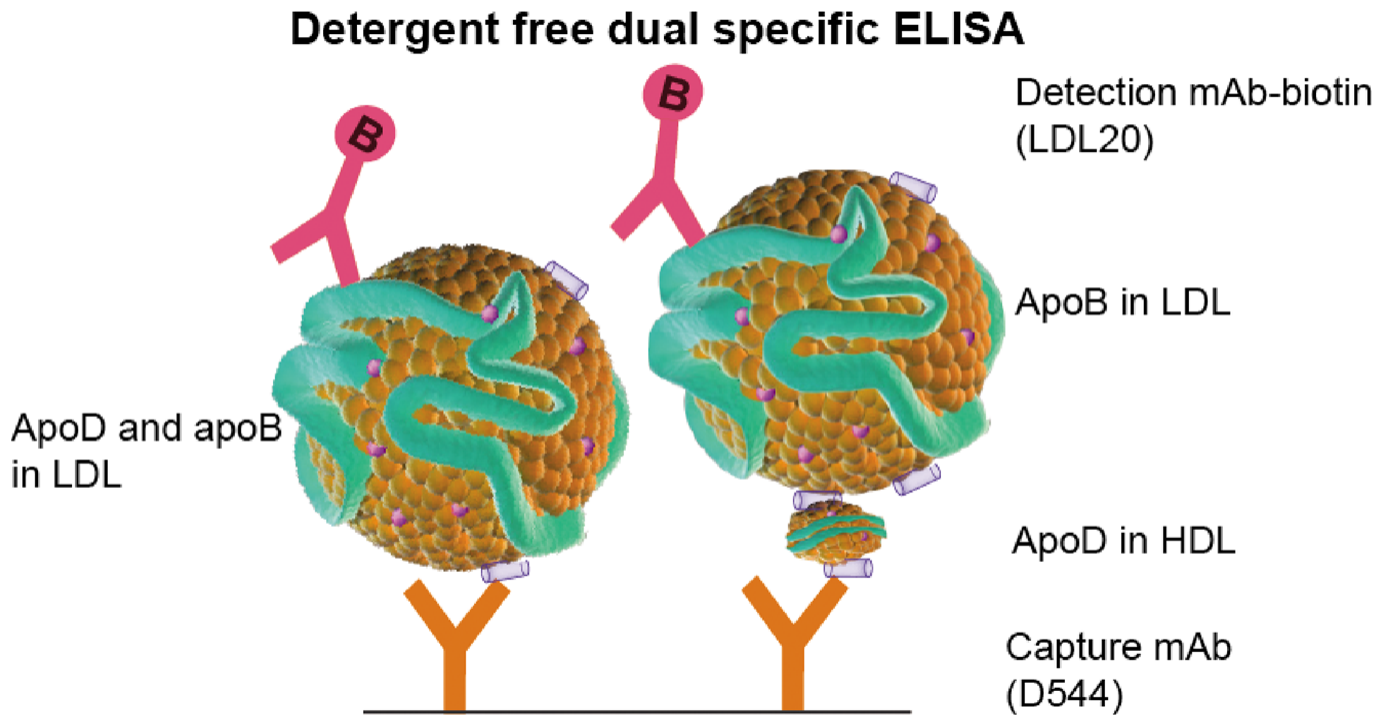
Detergent-free dual-specific ELISA. The absence of detergent maintains the structure of intact lipoprotein particles. The particles are immobilized using one specific antibody and the content is detected by using another.

ApoD in plasma is found primarily in HDL particles and to a lesser extent in LDL and VLDL particles. To investigate the presence of particles containing both apoD and apoB, we performed a dual-specific ELISA using the anti-apoD mAb D554 as a capture antibody and a biotinylated anti-apoB mAb, LDL20, for detection. To avoid disturbing the integrity of the lipoprotein particles, the ELISA was performed in the absence of detergents. Using this approach, we could detect not only lipoprotein particles carrying both apoD and apoB but also potential complexes between HDL and LDL or VLDL particles, as depicted in Fig. 1.

**Figure 2:**
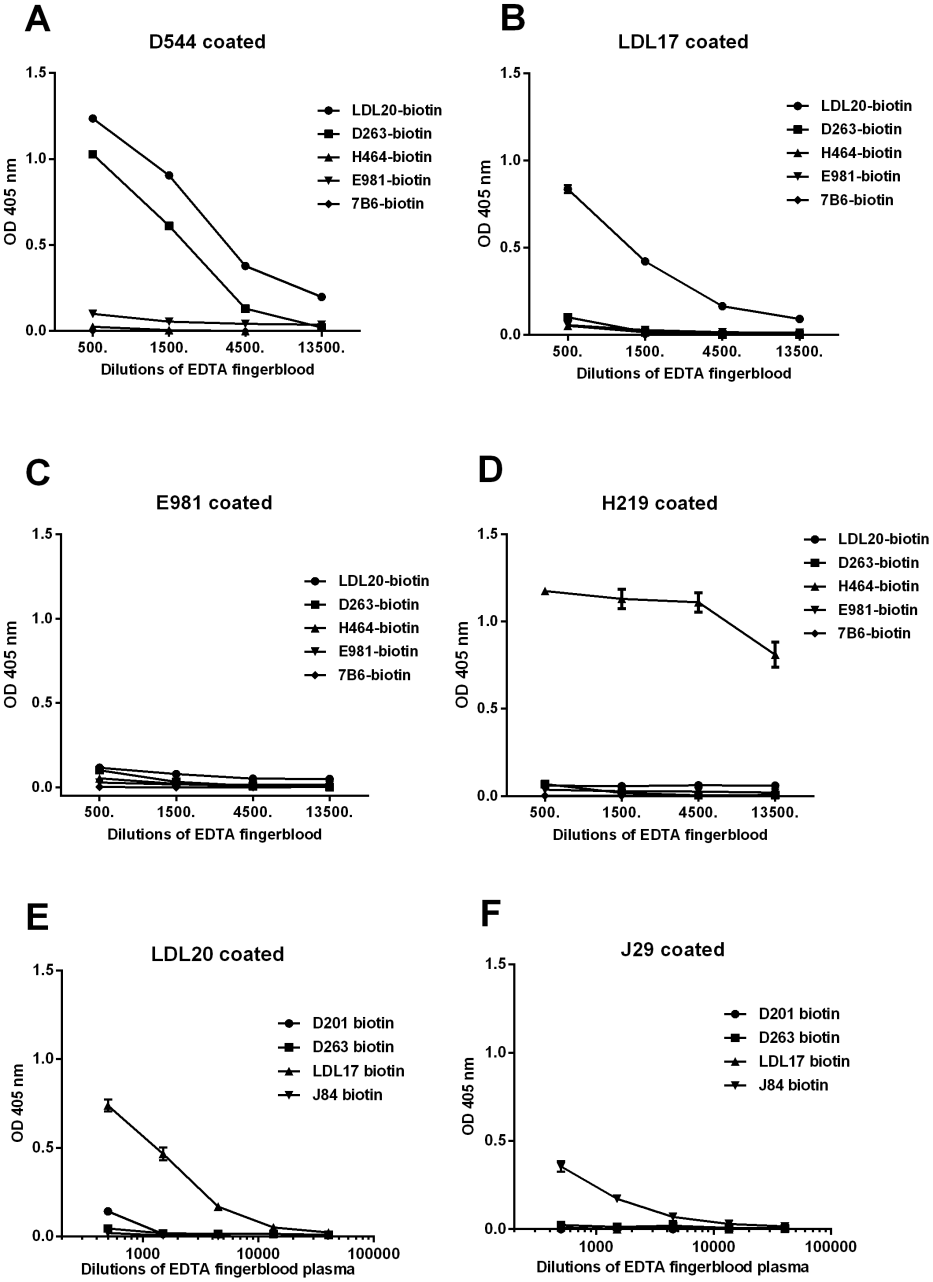
Anti-apoD antibodies capture ApoB in a detergent-free dual specific ELISA. The following capture antibodies functional under detergent-free conditions were used: A) anti-apoD (D544), B) anti-apoB (LDL17), C) anti-apoE (E981), D) anti-apoH (H219), E) anti-apoB (LDL20) and F) anti-apoJ (J29). Human EDTA finger blood plasma, prepared as described in the methods, was diluted with PBS containing 0.1% BSA. Detecting antibodies in A–D were LDL20-biotin (anti-apoB), D263-biotin (anti-apoD), H464-biotin (anti-apoH), 7B6-biotin (anti-IFNγ) and E981 (anti-apoE), and in E and F were D201-biotin (anti-apoD), D263-biotin (anti-apoD), LDL17-biotin (anti-apoB) and J84-biotin (anti-apoD). Note that E981 was also used for capture in C). The means ± SD of four replicates are shown. Results within each panel are from the same donor. Experiments A–D were repeated three times using the same donor, and experiments E and F were repeated four times using four different donors.

The dual-specific apoD/B ELISA analysis of plasma from finger blood yielded a surprisingly strong signal that was higher than that seen when immobilized apoD was probed with the anti-apoD antibody, D263-biotin (Fig. 2A). Similar results were obtained when performing the dual-specific ELISA with two other anti-apoD capture antibodies, D201 and D263 (data not shown). Similarly, the detection mAb LDL20-biotin in Fig. 2A could be replaced by a different anti-apoB antibody, LDL17-biotin, with comparable results (S2 Figure). Furthermore, diluting the plasma yielded very similar titration curves from the dual-specific ELISA as the same plasma from an apoB-specific ELISA (Fig. 2B). As apoD has been reported to be scarce in LDL and VLDL particles, much of this signal is likely attributable to the recognition of complexes between apoD-containing HDL and apoB-containing LDL (or VLDL) particles. Using the same type of dual-specific ELISA with antibodies against apoE (Fig. 2C), apoH (Fig. 2D) or apoJ (Fig. 2E) for capture resulted in weak or absent signals.

**Figure 3:**
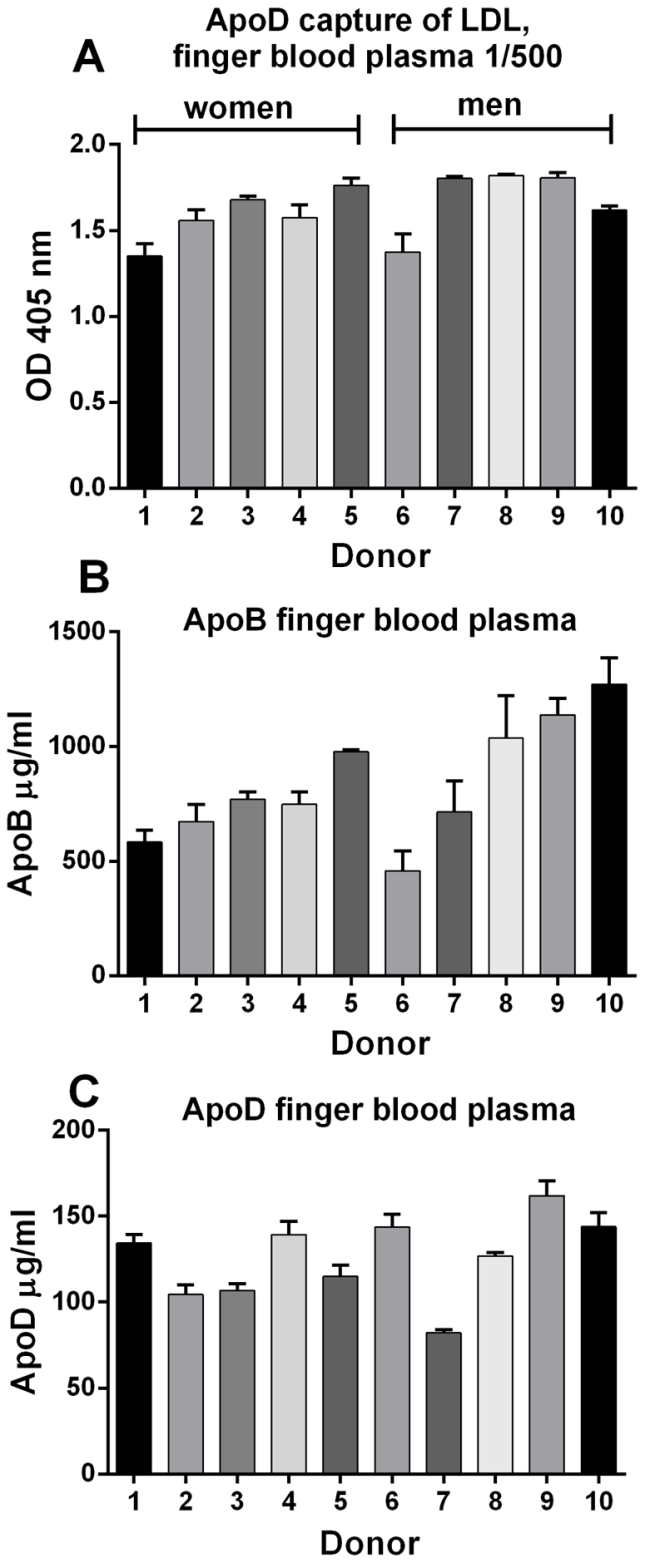
The interaction between apoD and apoB is a general mechanism. Finger blood from ten donors was assayed for A) apoD/apoB interaction in a detergent free dual-specific ELISA using D544 for capture and biotinylated LDL20 for detection B) ELISA for apoB levels and C) ELISA for apoD levels. Note that finger blood plasma, in figures B and C, was arbitrary calculated as half the finger blood volume, with the remaining volume assumed to be cells. Of the ten donors, No. 1–5 were women, and No. 6–10 were men, all between 30–62 years old. Means ± SD of four replicates are shown.

To demonstrate the presence of these putative lipoprotein complexes in the general population, finger blood from ten healthy volunteers was tested using the apoD/B ELISA, and as seen in Fig. 3A, all samples displayed ELISA values comparable to those seen in Fig. 2. For reference, the apoB and apoD levels in the same samples were measured separately (Fig. 3B and 3C).

**Figure 4:**
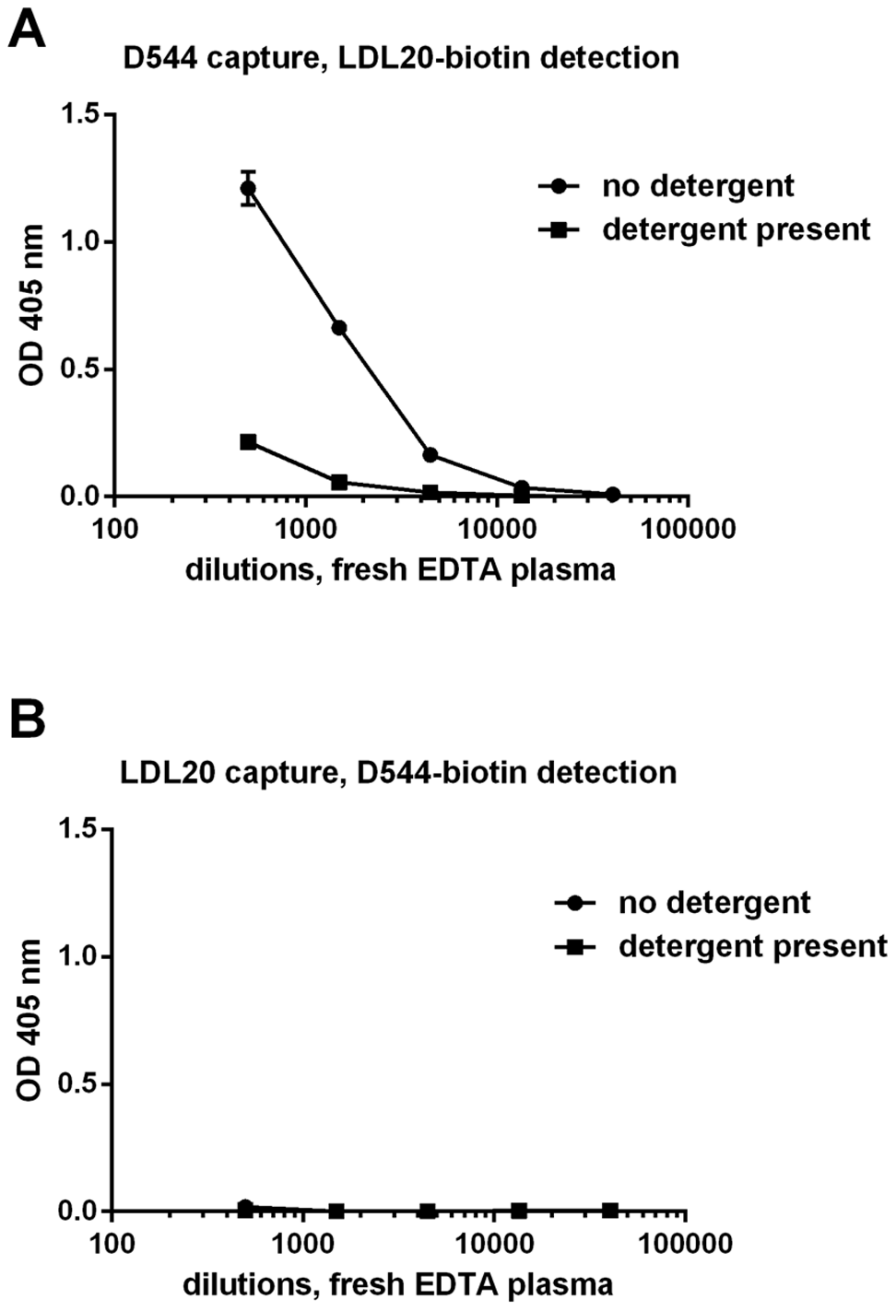
Binding between apoB and apoD is disturbed by detergents and is dependent on the immobilized antigen. Finger blood plasma was treated with or without detergent before being added to ELISA plates. A) anti-apoD (D544) was used as the coating antibody, allowing for multiple apoD bonds to each LDL particle, as detected using anti-apoB (LDL20-biotin). B) LDL20 was used as the coating antibody, and biotinylated D544 was used as the detection antibody. The means ± SD of four replicates are shown. Experiments were repeated three times with same donor.

To determine the importance of having intact lipoprotein particles, we repeated the dual-specific apoD/B ELISA in the presence of detergent, using anti-apoD-D544 as the capture mAb and anti-apoB-LDL20 as the detecting mAb. As seen in Fig. 4A, only small amounts of apoB bound to D544 when detergent was present, indicating that intact lipoprotein particles are needed for the apoD-LDL interaction. More surprisingly, when we reversed the system, using LDL20 as the capture antibody and biotinylated anti-apoD D544 as the detecting antibody, no complexes could be detected, independent of the presence of detergent (Fig. 4B).

### 3.2 ApoD is a key factor in the interaction between lipoprotein particles

To further investigate the potential interaction between apoD-containing particles and LDL and the role of apoD in this interaction, we used both purified apoD and isolated HDL preparations in combination with purified LDL in the same dual-specific ELISA. ApoD was purified from plasma by affinity chromatography in the presence of detergent, and a single band corresponding to apoD was observed on SDS-PAGE (S1 Figure). We also used HDL2 and HDL3 particles, which differ both in size (HDL2 is significantly bigger) and apoD content (HDL3 contains more apoD). The apoD and apoB content in the different preparations was determined by ELISA, confirming the differences in apoD content (Table I). HDL2 contained some apoB, which often happens when HDL2 is contaminated with small dense LDL.

**Table 1:** ApoB and ApoD content in lipoprotein particles.

	ApoB (ng)	ApoD (ng)
HDL2 (1 µg)	106	12.9
HDL3 (1 µg)	ND*	68.9
LDL (1 µg)	1290	7.9
VLDL (1 µg)	280	6.1
ApoD (100 ng)	ND**	100

ND*: not detected; the amount was lower than 0.12 ng/1 µg HDL3

ND**: not detected; the amount was lower than 0.03 ng/100 ng apoD.

ApoD was purified from human EDTA-plasma.

**Figure 5:**
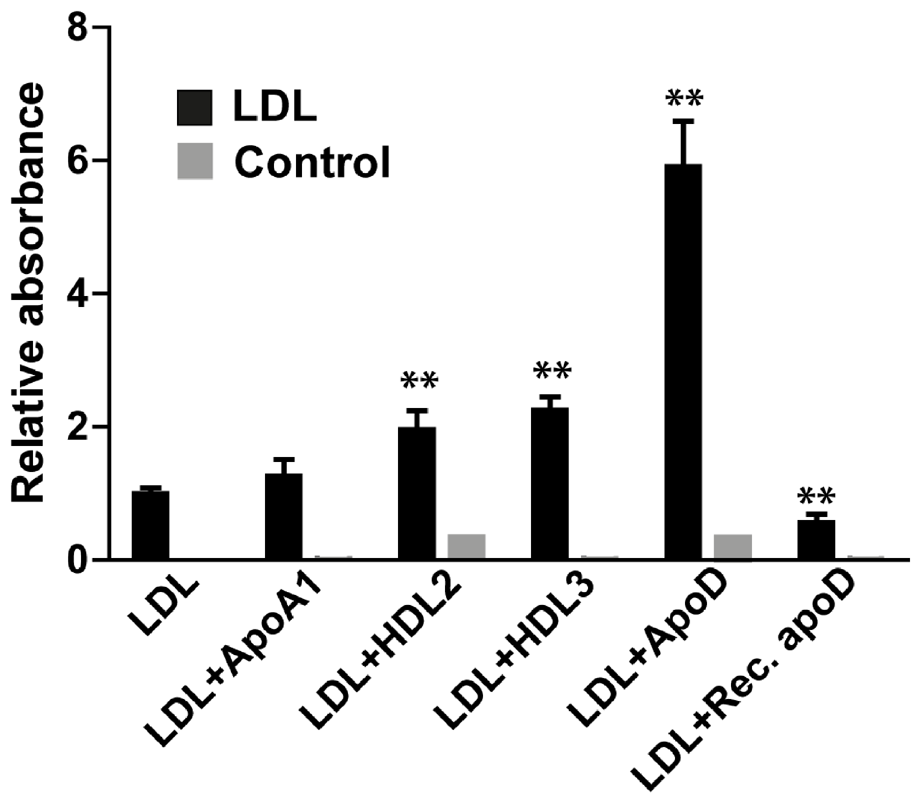
HDL3 and purified apoD facilitates the binding of LDL to D544. Anti-apoD (D544) was used as the capture antibody and anti-apoB (LDL20-biotin) was used as the detection antibody in a detergent free dual-specific ELISA. LDL (333 ng/ml) was added either alone or in combination with ApoA1 (100 ng/ml), HDL2 (1000 ng/ml), HDL3 (1000 ng/ml), apoD (native) (100 ng/ml) or recombinant apoD (100 ng/ml). As a the control, LDL was omitted, but the same amounts of apoA1, HDL2, HDL3, apoD or recombinant apoD were added. The control without LDL is shown in grey bars. Relative absorbance was calculated by dividing the absorbance value by the absorbance of LDL alone. The experiment was repeated 6 times, each time using four replicates, and mean of the 6 experiments ± SD is shown. ** p<0.01 compared with LDL alone; p-values were calculated using the Mann-Whitney test with GraphPad prism 6.

In Fig. 5, purified LDL alone and LDL co-incubated with HDL2, HDL3 or purified apoD were analyzed via the dual-specific ELISA. As expected, LDL itself gave a weak to moderate positive signal due to the concomitant presence of apoD and apoB in some of these particles. This signal was roughly doubled by the addition of either HDL2 or HDL3, while addition of purified native apoD led to a 6-fold binding enhancement. Interestingly, recombinant apoD, in which 5 amino acids had been removed to reduce the hydrophobicity, did not promote LDL binding.

**Figure 6:**
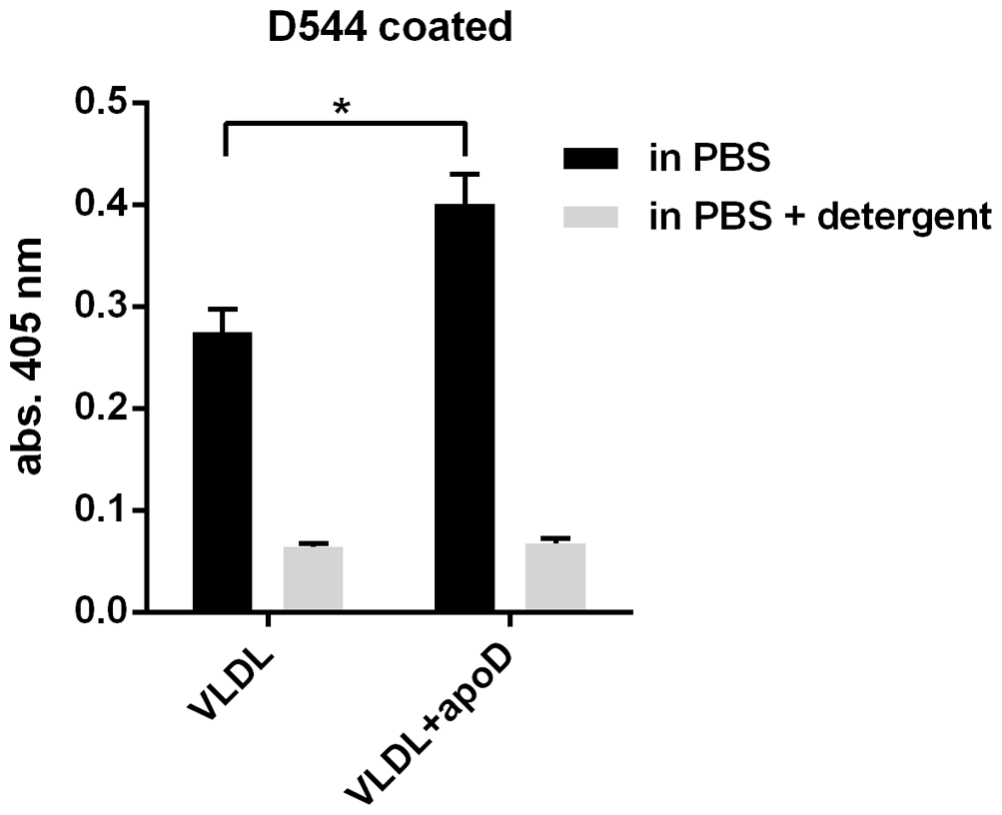
VLDL is immobilized by D544. The effect of adding extra apoD (100 ng/ml) to VLDL (1000 ng/ml) was analyzed using a dual-specific ELISA with or without detergent present. The capture antibody was anti-apoD (D544), and the detection antibody was anti-apoB (LDL20-biotin). The means ± SD of four replicates are shown. Experiments were repeated three times. * p<0.05; p-values were calculated using the Mann-Whitney test with GraphPad prism 6.

Purified VLDL also binds to anti-apoD in a detergent-sensitive manner and the binding is also enhanced by the addition of extra apoD (Fig. 6). However, the binding gives lower values and these values are not raised as much by addition of purified apoD.

### 3.3 Biosensor analysis of lipoprotein interactions

**Figure 7:**
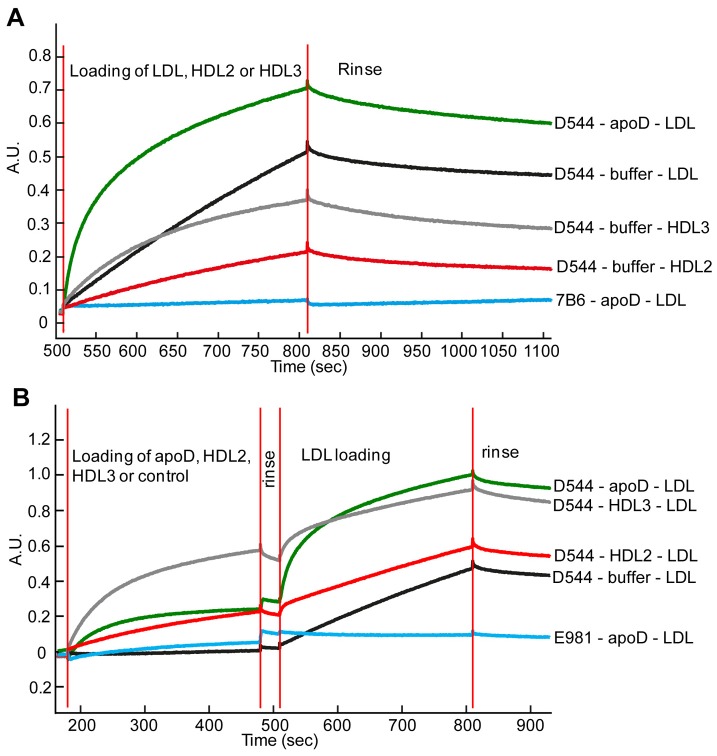
Biosensor monitoring of apoD-mediated binding of LDL. In the Blitz biosensor analysis, the signal corresponds to the mass collected on the biosensor surface. Here we used Streptavidin-coated sensor surfaces that were loaded with biotinylated antibody (10 µg/ml for 120 seconds) followed by apoD (2 µg/ml for 300 seconds) or buffer-control (300 seconds). The surfaces were subsequently loaded with the LDL, HDL2 or HDL3 lipoprotein particles (100 µg/ml for 300 seconds). Between loading reagents, the sensors were rinsed for 30 seconds. The buffer used for all steps and dilutions was PBS, azide, 0.1% BSA. **A**) The green, black, grey and red lines represent experiments preloaded with anti-apoD D544. The blue line indicates the control preloaded with isotype control antibody 7B6 (anti-IFNγ). The green and blue lines indicate that ApoD was preloaded, while the black, grey, and red lines indicate the buffer controls. The panel shows the final step in which the lipoprotein particles bind to the antibody loaded with or without apoD. The green, black and blue lines show binding of LDL, the grey line shows binding of HDL3 and the red line shows binding of HDL2. **B**) The binding step of the biotinylated antibody was omitted. Green, black, red and grey lines indicate experiments that used D544-biotin, and the blue line indicates that E981-biotin (anti-apoE) was used. Subsequent reactions show the addition of HDL2 in red, HDL3 in grey, apoD in green and blue, or buffer in black to the preloaded antibodies. In the last step, LDL was loaded in all the conditions.

To measure binding in a more direct manner, we also used biosensor technology. We were able to not only register binding but also obtain information about the relative association affinities of the interactions. In agreement with the previous ELISA results, the anti-apoD mAb D544 bound purified LDL due to the presence of apoD in some of the LDL particles. (Fig. 7A). However, LDL binding was considerably faster and stronger when the D544 mAb was first loaded with purified apoD. The mAb D263 yielded similar results (S3 Figure).

As expected, the antibody also bound HDL3 and HDL2 to an extent reflecting the amount of apoD in the respective particle. Similar to the enhanced LDL binding observed in the presence apoD, preloading the D544 mAb with HDL3, and to some extent with HDL2, also increased the initial speed of LDL binding, though this effect was less strong compared to using purified apoD (Fig. 7B).

### 3.4 ApoD promotes HDL binding to cells

**Figure 8:**
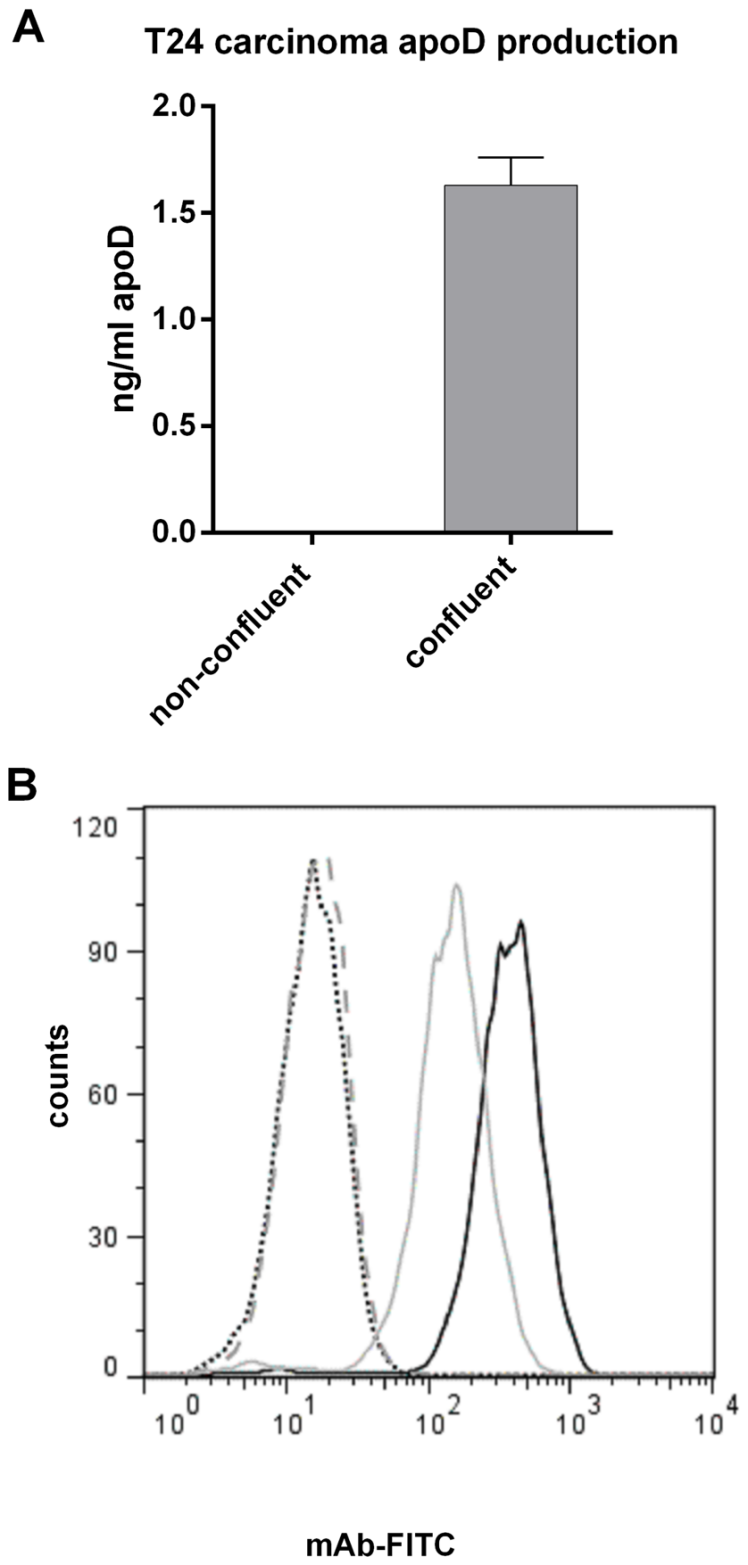
ApoD production in T24 cells was assayed by ELISA for culture supernatant and by FACS for intracellular staining. **A**) T24 cells were grown in DMEM with 10% FBS in 25 cm^2^ flasks. Three flasks each of confluent cells (2.5 million in 9 ml) and non-confluent cells (1.5 million in 7 ml) were sampled 4 times. ApoD content was assayed by apoD ELISA; detection limit  = 0.1 ng/ml. **B**) D544 (anti apoD) was used to stain confluent (black) and non-confluent (grey) T24 carcinoma cells. The isotype control is depicted with a dotted black line for confluent T24 cells and with a dashed grey line for non-confluent T24 cells. Experiments were repeated three times.

**Figure 9:**
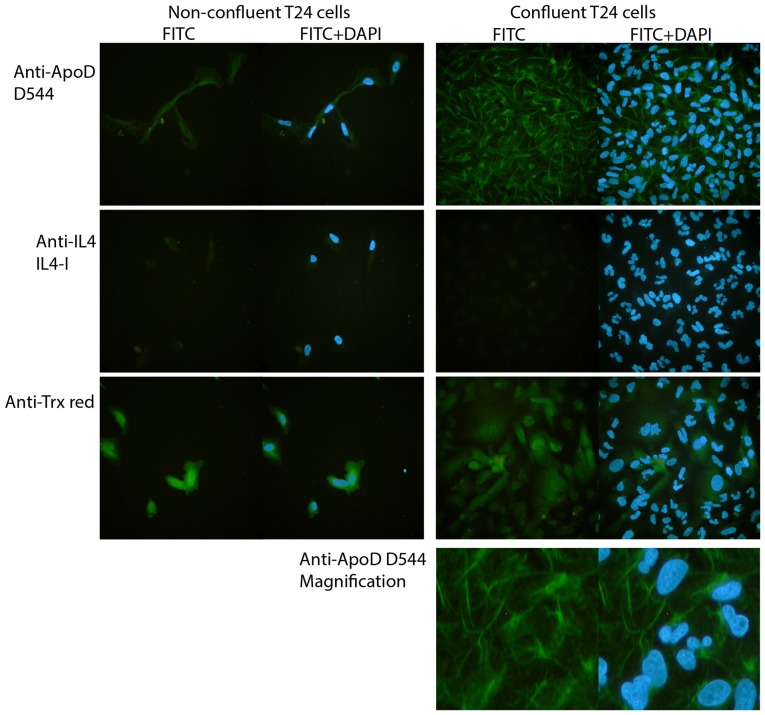
Non-confluent T24 cells stain positive for apoD. Cells were seeded on objective glasses in petri dishes. After either 2 days (non-confluent) or 9 days (confluent), the cells were washed and incubated with a negative isotype-control mAb (anti-IL4), a positive control anti-thioredoxin reductase mAb (anti-Trxred), or anti-apoD D544 mAb. Bound monoclonal antibodies were stained with polyclonal anti-mouse FITC-conjugated Fab fragments. Nuclei were visualized with DAPI. Cells were photographed with a Leica DMRB fluorescent microscope (shutter speed FITC 2 sec, and DAPI 1/30sek). Staining was repeated three times.

As apoD is expressed by many cell types, we also investigated the interaction between apoD and lipoprotein particles in a cellular setting. For this we used the urinary bladder carcinoma cell line T24, which produces apoD and displays contact inhibition when cultured in vitro, which has previously been shown to affect apoD production [25], [26], [27], [28], [29]. In agreement with this latter finding, apoD production was high in confluent, growth-arrested T24 cell cultures (2,5 million cells in 9 ml), but was not detectable in non-confluent proliferating cultures (1,5 million cells in 7 ml) (Fig. 8A). However, despite the absence of apoD in the supernatant of proliferating cells, apoD was abundant intracellularly, as shown by flow cytometry (Fig. 8B) and by immunocytochemistry (Fig. 9).

**Figure 10:**
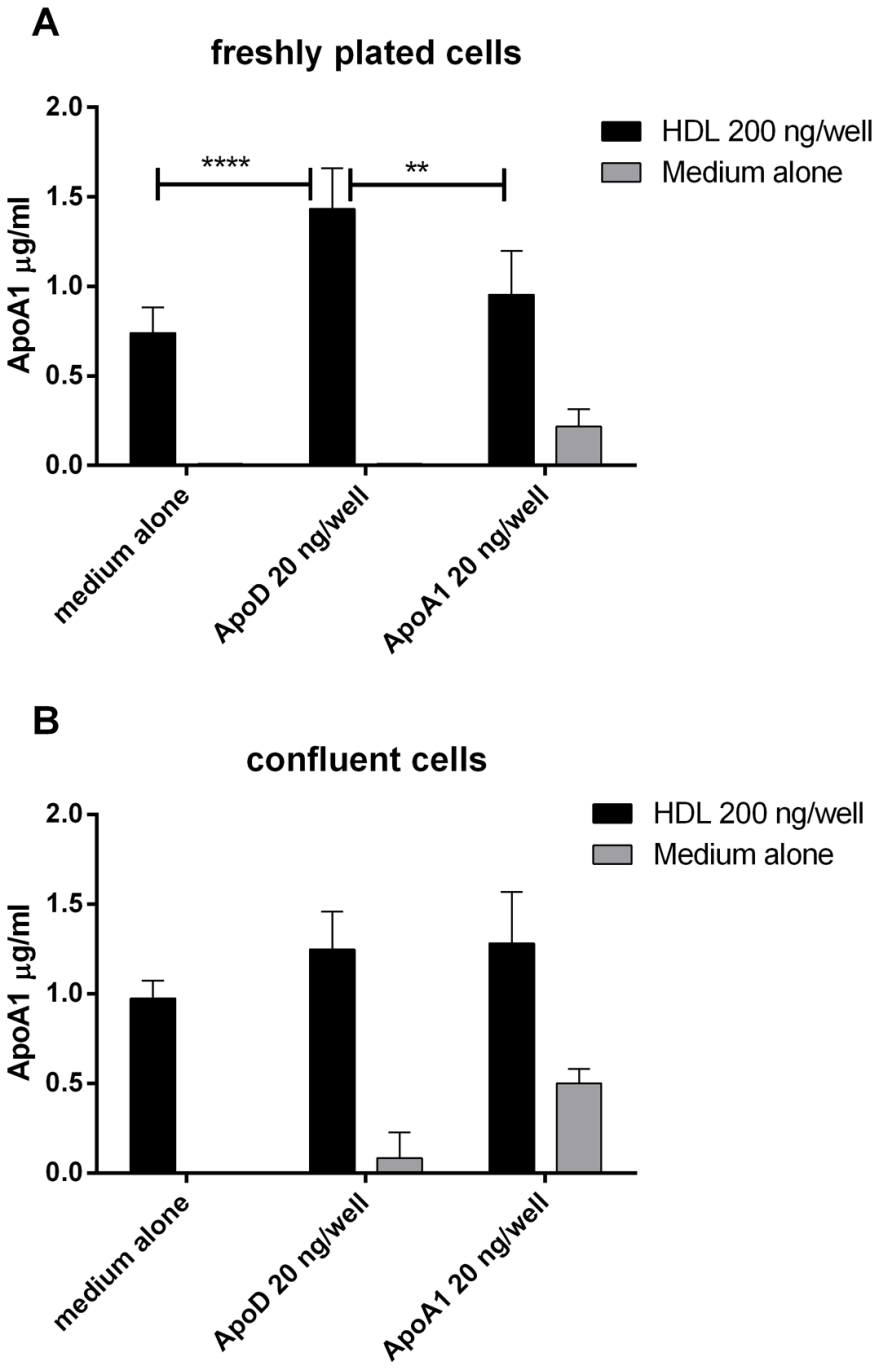
ApoD facilitates binding of HDL to growing T24 cells. T24 cells grown in 24-well plates were washed with protein-free RPMI two times, and 200 µl/well RPMI supplemented with 0.1%BSA was added with or without HDL (total HDL, 200 ng/well) and with or without apoD (20 ng/well) or apoA1 (20 ng/well). After incubation for 1 hour at 21°C, the plate was washed three times with protein-free RPMI, and bound apoA1 was released using detergent and assayed by apoA1 ELISA. In **A**) the cells were non-confluent and in **B**) the cells were confluent. Means ± SD of four experiments are shown. ** p<0.01, ****p<0.0001; p-values were calculated using two-way ANOVA with Holm-Sidal multiple comparison test, GraphPad Prism 6.

To measure potential interactions between cells and lipoprotein particles, cells were incubated with HDL in the presence or absence of apoD. After removing unbound material by washing, the bound particles were solubilized, and the apoA1 (HDL) content in the soluble fraction was analyzed by ELISA. As demonstrated in Fig. 10, significant amounts of HDL were bound to both the confluent and non-confluent cultures. However, addition of purified apoD to the non-confluent, proliferating cells significantly promoted the binding of HDL, whereas the same addition to confluent cells only had a marginal positive effect. Similar experiments with LDL showed that apoD did not help LDL bind to the cells (data not shown).

## Discussion

We have here shown that apoD, immobilized by specific monoclonal antibodies on a solid surface, mediates the binding of LDL to the surface. We used a dual-specific ELISA in which antibodies to apoD bound intact apoB-containing lipoproteins equally efficient as antibodies specific for apoB. This property was unique for apoD and was not seen with other exchangeable apolipoproteins such apoE, apoH and apoJ, all of which are present in HDL and LDL/VLDL lipoprotein particles [32], [33], [34]. Adding detergent inhibited the apoD-mediated binding of apoB, demonstrating that changes in the LDL structure affect the formation of the complex. Surprisingly, LDL/VLDL particles immobilized by antibodies to apoB could only marginally capture apoD, suggesting that the affinity of a single apoD is not sufficient to withstand the repeated washings and incubations of the ELISA protocol. Therefore, several apoD molecules will likely be needed to achieve sufficient avidity to immobilize LDL to a surface via apoD-binding. This moderate affinity between apoD and LDL is likely suitable for the rapid encounters between HDL-containing apoD and LDL particles in the plasma, as a high affinity interaction could yield more stable complexes and aggregation. Notably, adding purified apoD helped D544 to bind more LDL, indicating that the apoD contact with LDL is a very rapid event. Hydrophilized recombinant apoD, on the other hand, was not capable of binding LDL, probably because some of the hydrophobic amino acids required for binding have been removed in the recombinant apoD. These results emphasize the need to use native apoD for functional studies of this lipocalin.

Biosensor analysis is a nice complement to ELISA as it measures binding in a direct way. Mass binding to the sensor-tip results indiscriminately in a stronger signal, and the speed of binding can be monitored and is proportional to the affinity/avidity of the reaction. We demonstrate that although LDL contains some apoD and will bind to anti-apoD (D544), the binding was considerably faster if the D544 antibodies were preloaded with apoD. In addition, HDL3 bound both more quickly and with more mass to D544-coated surfaces than did HDL2. As mentioned above, HDL3 has a higher density than HDL2 but is also considerably smaller and carries more apoD. Preloading the surface with purified apoD is the most efficient way to tie down LDL, but HDL3 also yields a high initial speed of LDL binding compared to D544 alone or loaded with HDL2. One explanation for the difference between the quick binding of LDL to apoD- or HDL3-coated surfaces and the slower linear binding of LDL to D544 alone is that, in the latter case, apoD needs to reposition to allow multiple bonds between the surface and LDL. In animal experiments, apoD was shown to be important in protecting against oxidative stress [6], [7], [24]. Interestingly, HDL3 has been reported to be more important than HDL2 for protection against LDL-copper-catalyzed oxidation [35], [36]. The biosensor results obtained here show that HDL3 bound LDL more avidly, supplying a potential explanation for the protection of LDL by HDL3.

T24 carcinoma cells display contact inhibition if the cells are washed with fresh medium after reaching confluency. Washing removes growth factors and ensures that the cells are kept below the threshold needed to overcome contact inhibition [37]. Interestingly, purified apoD may aid growing T24 cells in binding HDL, but not contact-inhibited, growth-arrested cells, indicating a possible growth-regulated receptor for apoD. Additionally, only supernatants from growth-inhibited T24 cells contained detectable amounts of apoD. These results agree with several reports showing that apoD production increases upon growth arrest . However, intracellular staining with D544 revealed that non-confluent T24 cells contained substantial amounts of apoD, albeit less than confluent cells. The reason for these unexpected results may be that non-confluent cells produce, but do not export, apoD. Another possibility is that non-confluent cells secrete apoD but then internalize it efficiently via receptors not expressed by quiescent cells. One might speculate that apoD plays a role in the lipid efflux to and from T24 cells. A putative mechanism is that apoD helps to supply lipids to growing cells, presumably via an apoD-recognizing receptor. Once contact inhibition is induced, the cells downregulate this receptor and secrete surplus lipids via apoD-containing lipoproteins. However, verifying such a mechanism would require extensive studies to find an apoD binding receptor.

Large amounts of apoD accumulate at sites of nerve injury [8]. Human genetic studies and studies in apoD knockout mice have revealed that apoD is important not only for brain and nerve functions but also for controlling inflammation and macrophage homeostasis and to maintain a normal lipid profile [7], [20]. Here we propose that apoD acts a mediator/cofactor in the interaction of HDL particles with LDL particle and with cells. ApoD would presumably anchor HDL to LDL or cells with an affinity/avidity that result in a suitable on-off rate for lipid trafficking. This mechanism could partly explain several of the physiological roles of apoD. For example, apoD has been reported to help regulate LCAT activity [21], [22], [23]. If apoD is an important mediator of lipid trafficking, one might speculate that the intense up-regulation of apoD at the site of nerve injury is necessary to attract and increase the local concentration of HDL particles for lipid transport between glial cells or macrophages and nerve cells and Schwann cells.

ApoD is an enigmatic protein, but the results of the current study reveal an important function of apoD as a mediator of the interaction between HDL particles to LDL particles and to growing cells. However, further studies are needed to fully elucidate the physiological function of apoD. Several important questions remain, such as determining whether other cofactors are necessary for these lipoprotein interactions and how the lipid hormone-carrying role of apoD affects these interactions. Will the lipid load of this lipocalin change its function? Identifying and characterizing the potential cellular receptor for apoD and how it is regulated during cell growth will also be necessary.

## Supporting Information

S1 Figure **Affinity purified apoD.** The Mabtech ApoD standard was purified by affinity purification from EDTA aprotinin plasma in the presence of a non-ionic detergent. Approximately 0.5 µg was analyzed by SDS-PAGE in the presence of a reducing agent and stained with SimplyBlue, Invitrogen.
(TIF)

S2 Figure **LDL/VLDL captured by anti-apoD can be detected by either LDL20-biotin or LDL17-biotin.** D544 was used to capture lipoproteins from human EDTA finger blood plasma, prepared as described in the methods. Biotinylated detection antibodies were LDL20 (anti-apoB), and LDL17 (anti-apoB). D263-biotin was used as a positive control. Means ± SD of three replicates are shown.
(TIF)

S3 Figure **Biosensor monitoring of apoD-mediated binding of LDL.** In the Blitz biosensor analysis, the signal corresponds to the mass collected on the biosensor surface. Here we used Streptavidin-coated sensor surfaces that were loaded with either D263-biotin or the isotype control 7B6-biotin (10 µg/ml for 120 seconds). Sensors were rinsed (30 seconds) and loaded with apoD (2 µg/ml) or buffer-control (120 seconds), and then rinsed again and loaded with 100 µg/ml LDL (120 seconds).
(TIF)
