# Supplementary figures and images for: ApoD Mediates Binding of HDL to LDL and to Growing T24 Carcinoma
Source: PLoS One. 2014 Dec 16;9(12):e115180. doi: 10.1371/journal.pone.0115180 (PMC4267786; doi:10.1371/journal.pone.0115180)

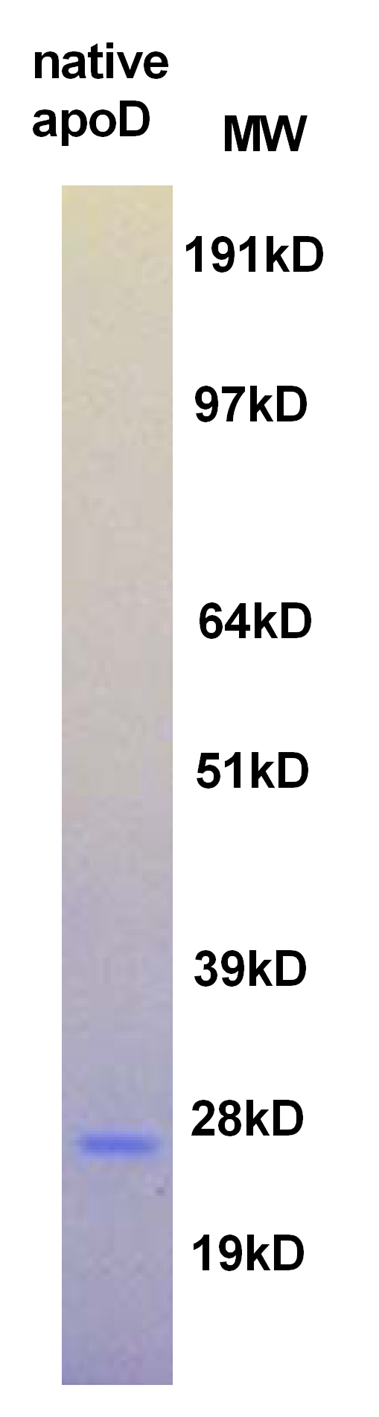

Supplement: S1 Figure — Affinity purified apoD. The Mabtech ApoD standard was purified by affinity purification from EDTA aprotinin plasma in the presence of a non-ionic detergent. Approximately 0.5 µg was analyzed by SDS-PAGE in the presence of a reducing agent and stained with SimplyBlue, Invitrogen. (TIF) [file pone.0115180.s001.tif]

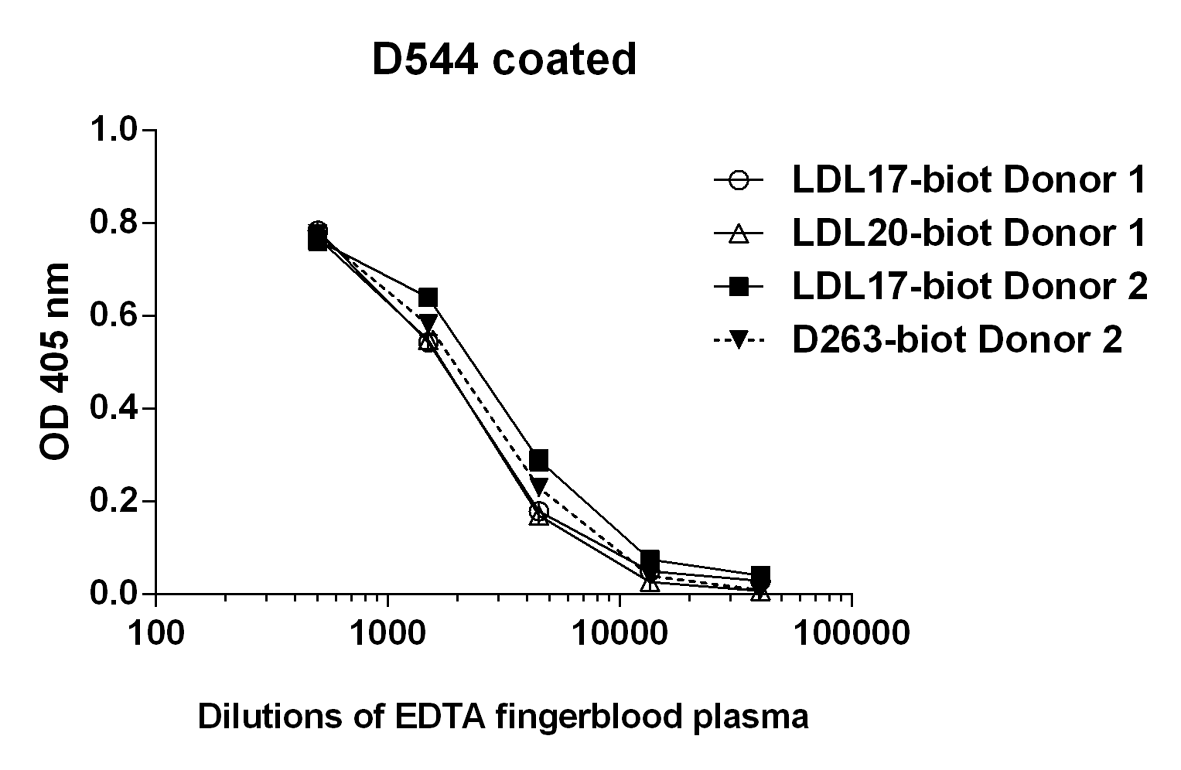

Supplement: S2 Figure — LDL/VLDL captured by anti-apoD can be detected by either LDL20-biotin or LDL17-biotin. D544 was used to capture lipoproteins from human EDTA finger blood plasma, prepared as described in the methods. Biotinylated detection antibodies were LDL20 (anti-apoB), and LDL17 (anti-apoB). D263-biotin was used as a positive control. Means ± SD of three replicates are shown. (TIF) [file pone.0115180.s002.tif]

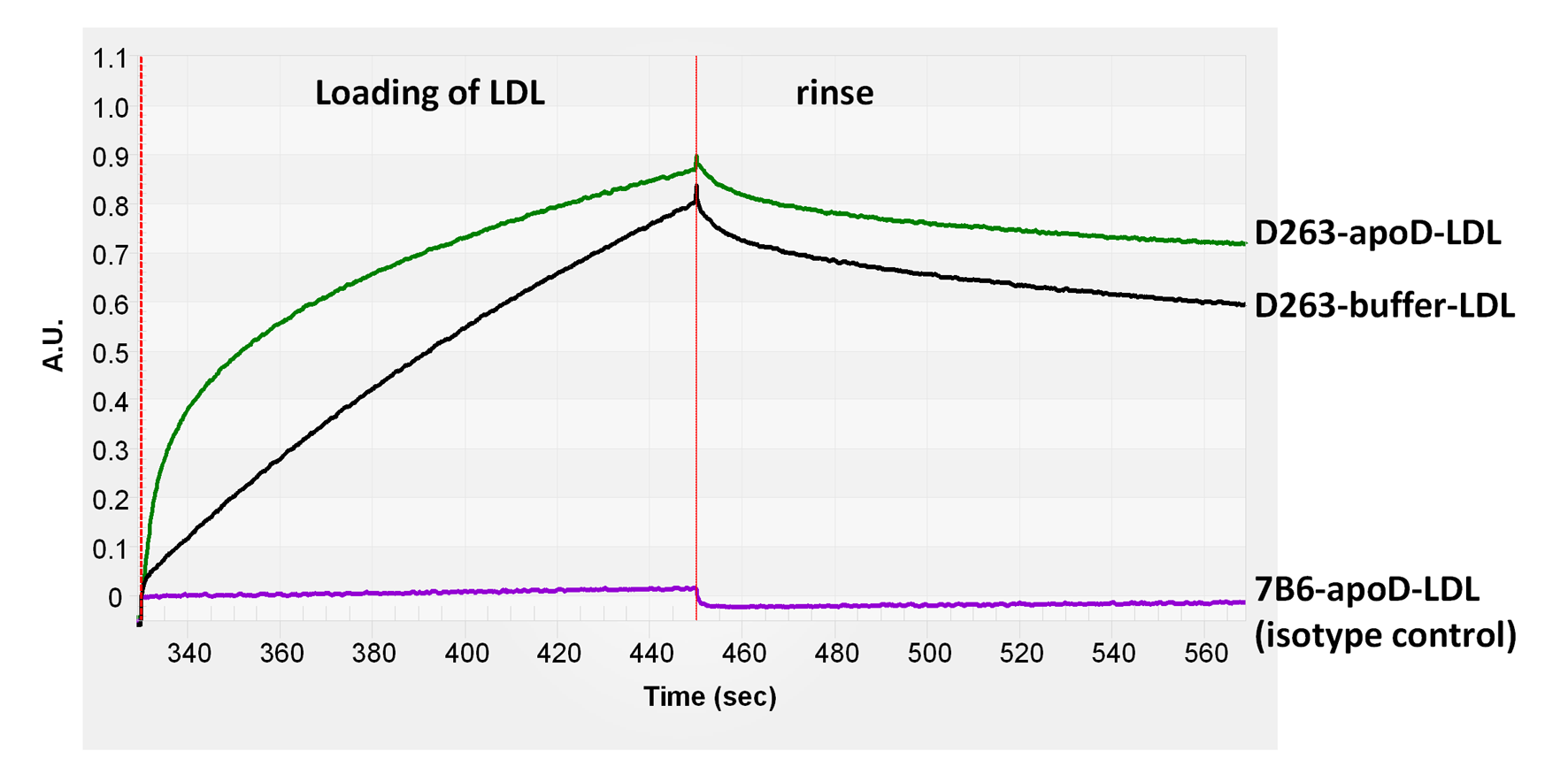

Supplement: S3 Figure — Biosensor monitoring of apoD-mediated binding of LDL. In the Blitz biosensor analysis, the signal corresponds to the mass collected on the biosensor surface. Here we used Streptavidin-coated sensor surfaces that were loaded with either D263-biotin or the isotype control 7B6-biotin (10 µg/ml for 120 seconds). Sensors were rinsed (30 seconds) and loaded with apoD (2 µg/ml) or buffer-control (120 seconds), and then rinsed again and loaded with 100 µg/ml LDL (120 seconds). (TIF) [file pone.0115180.s003.tif]
